# Descemet Membrane Endothelial Keratoplasty and Descemet Stripping Only Using a 3D Visualization System

**DOI:** 10.3390/jcm12175746

**Published:** 2023-09-04

**Authors:** Antonio Cano-Ortiz, Álvaro Sánchez-Ventosa, Vanesa Díaz-Mesa, Timoteo González-Cruces, Marta Villalba-González, Alberto Villarrubia-Cuadrado

**Affiliations:** Department of Ophthalmology, Hospital La Arruzafa, 14012 Córdoba, Spainvdiaz@hospitalarruzafa.com (V.D.-M.); timoteogc@gmail.com (T.G.-C.); marta.villalba7@gmail.com (M.V.-G.); alvillarrubia@yahoo.com (A.V.-C.)

**Keywords:** Descemet membrane endothelial keratoplasty, Descemet stripping only, 3D visualization, Fuchs’ endothelial corneal dystrophy

## Abstract

**Highlights:**

**What is known?**

- The 3D visualization system allows the surgeon to operate in a more comfortable position than a conventional microscope.
- Looking at the surgical field on a large, high-definition 3D screen has advantages for surgical assistance and instructional purposes.

**What is new?**

- The 3D visualization system can be successfully used for DSO procedures.
- Learning curve is short, as there is no training time required for successful use of 3D visualization system.

**Abstract:**

(1) Purpose: The aim was to analyze the outcomes of Descemet’s membrane endothelial keratoplasty (DMEK) and Descemet stripping only (DSO) surgeries using a glasses-assisted NGENUITY^®^ 3D visualization system (Alcon Laboratories, Fort Worth, TX, USA). (2) Methods: Five consecutive cases of DMEK surgery and four consecutive cases of DSO were performed using the NGENUITY^®^ system in this prospective study carried out at the Arruzafa Hospital, Córdoba, Spain. Only one eye from each patient received surgery. Best corrected distance visual acuity (CDVA) using EDTRS charts, central corneal thickness using the Casia II optical coherence tomograph (Tomey Co., Nagoya, Japan), and endothelial cell count using the Tomey EM-4000 (Tomey Co., Nagoya, Japan) for DMEK cases or the Nidek CEM-530 (Nidek Co., Ltd., Gamagori, Japan) specular microscopes for DSO cases were recorded preoperatively and at 1 and 3 months postsurgery. (3) Results: DMEK cases included one male and four female subjects, with a mean age of 73.6 ± 9.5 years. Average improvement in CDVA 3 months after surgery was 0.46 ± 0.16 decimal. Average change in cell count between 1 and 3 months postsurgery was 360.75 ± 289.38 cells/mm^2^. DSO cases included four female subjects, with a mean age of 64.2 ± 9.7 years. The average improvement in CDVA 3 months after surgery was 0.09 ± 0.17 decimal. All cases also had phacoemulsification carried out. He average change in cell count between 1 and 3 months after surgery was 460 ± 515.69 cells/mm^2^. There were no associated complications during surgery or the follow-up period in any of the cases. (4) Conclusions: In addition to the known benefits of the use of a 3D visualization system during surgery, the present study shows that the system can be successfully used in both DMEK and DSO procedures with a very short learning curve for the surgeon.

## 1. Introduction

Descemet’s membrane endothelial keratoplasty (DMEK) is a partial-thickness corneal graft operation in which only Descemet’s membrane and the endothelium are replaced [1]. Descemet stripping only (DSO) has emerged as an alternative to DMEK for treating eyes with Fuchs’ endothelial corneal dystrophy (FECD) with healthy peripheral endothelium [2]. Rather than replacing endothelial cells with an allograft, DSO removes pathologic guttae via a central descemetorrhexis, allowing healthy peripheral endothelial cells to migrate into the central void and recover from the corneal edema.

DMEK procedures have traditionally been performed using standard surgical microscopes, but, more recently, three-dimensional (3D) visualization systems have also been employed. These systems were originally developed for vitreoretinal surgery [3,4], but their potential for anterior segment procedures has been explored over the last few years, with promising results in cataract [5], trabeculotomy [6], and endothelial procedures such as DMEK [7,8,9]. Performing surgery using these systems allows for an enhanced depth of field, high magnification and better positioning for the surgeon during surgery. Specifically for DMEK surgery, Panthier et al. [7] performed 12 consecutive cases of this type of surgery using the glasses-assisted NGENUITY^®^ 3D visualization system, version 1.4 (Alcon Laboratories, Fort Worth, TX, USA) and concluded that it is feasible; however, they found this more challenging, with the total time being longer compared with using a conventional microscope. They indicated that this method would be useful for instructional courses. Borroni et al. [8] evaluated the feasibility of DMEK tissue preparation using this system, concluding that it is feasible, with a slightly increased preparation time. They indicated that improved visualization allows a reduced staining time, which could be beneficial for eye banks because it may reduce the toxic effect of staining colorants. Recently, Morelli et al. [9], in a sample of 20 eyes operated with NGENUITY^®^ and 20 eyes using the traditional microscope, concluded that the main advantages of the heads-up approach may be the improved ergonomic comfort during surgery and the utility of assistants in surgical training.

The present study aims to describe the three-month outcomes of a series of clinical cases that underwent DMEK and DSO using the NGENUITY^®^ 3D visualization system.

## 2. Methods

**Figure 1:**
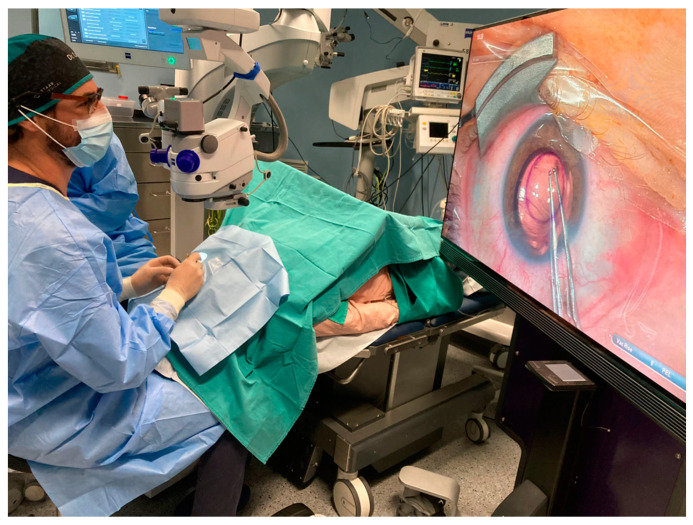
Image of the arrangement of the 3D visualization system during the surgery. The surgeon wears polarized glasses while looking at the large screen throughout the surgery.

All procedures were performed by the same experienced surgeon (ACO). The surgeon only received 1 session of training on the 3D visualization system. The 3D visualization system used was the NGENUITY^®^ 3D Visualization System. The system comprises three main components: a 3D high dynamic range camera, a 3D 4K organic light-emitting diode (OLED) display, and a high-speed graphics processor. An image of the system setup during surgery can be observed in Figure 1. Additionally, an OPMI-Lumera 700 surgical microscope (Carl Zeiss Meditec, Jena, Germany) with Callisto and intraoperative OCT was used to perform the surgeries; the Casia II Optical Coherence Tomography (OCT, Tomey Co., Nagoya, Japan) was used to determine the biometric parameters of the anterior segment, including anterior and posterior corneal surface topographies and thickness. Either the Tomey EM-4000 (Tomey Co., Nagoya, Japan) specular microscope for DMEK cases or the Nidek CEM-530 (Nidek Co., Ltd., Gamagori, Japan) specular microscope for DSO cases was used to carry out the endothelial cell count.

DMEK and DSO procedures were performed under locoregional anesthesia with peribulbar block.

## 3. DMEK Cases

Grafts for DMEK were provided by Arruzafa eye bank after having been stripped and placed on their sclerocorneal support. Grafts were immersed in 0.06% trypan blue dye (VisionBlue^TM^ 0.06%, D.O.R.C. International, Zuidland, The Netherlands) and trephined by the surgeon to the preferred width. DM was performed for the central 8.5 to 9 mm diameter using an inverted Price-Sinskey hook.

To visualize the Descemet membrane roll in the anterior chamber throughout the surgery, the graft was stained with 0.06% trypan blue solution (VisionBlue^TM^ 0.06%, D.O.R.C. International, Zuidland, The Netherlands) and inserted through the main incision with a glass injector (DMEK cartridge and injector kit; D.O.R.C. International, Zuidland, The Netherlands) into the recipient’s anterior chamber. The graft was placed endothelial side down (i.e., with the endothelial surface facing the iris) via indirect manipulation, balanced salt solution, and taping maneuvers. The graft was then gently unrolled, and 20% sulfur hexafluoride diluted with air (SF6) was injected underneath the graft to position it onto the recipient’s posterior stroma. The anterior chamber was left with complete SF6 fill for at least 25 min with the patient in strict supine position, followed by an SF6-liquid exchange to pressurize the eye while leaving an 80% SF6 bubble in the anterior chamber.

## 4. DSO Cases

A reverse Sinskey hook was used to lift off the edge of the Descemet membrane peripherally, and then DSO Gorovoy Forceps (#19097) (Moria SA, Paris, France) were used to perform the targeted sectorial descemetorhexis (5 mm), encompassing the entirety of the detached area.

The postoperative management for both groups included topical antibiotics given 4 times a day for the first 2 weeks and dexamethasone eye drops 4 times a day for the first month, which were then incrementally reduced over a 6-month period.

Five DMEK cases and four DSO cases were carried out by a single experienced surgeon using the NGENUITY^®^ system. Two of the DMEK and all of the DSO cases were combined with standard phacoemulsification. Patients were examined prior to surgery, and then 1 month and 3 months after surgery. Best corrected distance visual acuity (CDVA) using ETDRS charts recorded in decimal scale, endothelial cell count using specular microscopy, and corneal anterior segment biometric parameters using the OCT were determined at both postoperative follow-up visits.

## 5. Results

The five DMEK cases included one male and four female subjects, with a mean age of 73.6 ± 9.5 years. Only one eye was operated on from each patient. All patients had Fuchs’ dystrophy, except for one. Average values, standard deviations (SDs), and ranges for the different parameters in the preoperative period and at the different follow-up times postoperation are shown in Table 1.

One of the patients did not have pachymetry measurements obtained during the follow-up period, and, for the other two, there was no measurement in one of the follow-up visits.

**Figure 2:**
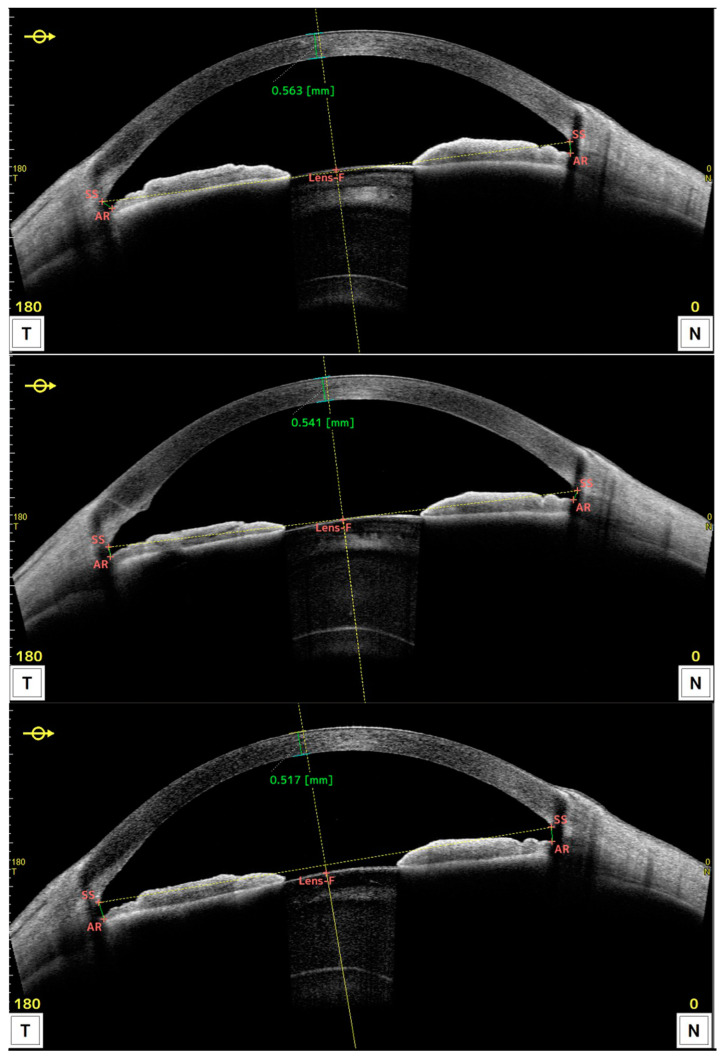
OCT along the horizontal meridian obtained preoperatively (**upper**), and 1 month (**middle**) and 3 months (**lower**) after DMEK surgery for one patient. Values shown in green refer to central pachymetry.

Figure 2 shows the OCT obtained preoperatively (upper), 1 month (middle), and 3 months (lower) after DMEK surgery for one patient, displaying central pachymetry values in green.

The average improvement in the CDVA 3 months after surgery compared with prior to surgery was 0.46 ± 0.16 decimal, although it must be noted that three of these cases also had standard phacoemulsification. Considering only those without phacoemulsification, CDVA improvement was 0.42 ± 0.20 decimal. The average change in cell count between 1 month and 3 months postsurgery was 360.75 ± 289.38 cells/mm^2^ (cell count was not obtained in the 1-month visit for one patient). There were no associated complications during the surgery or the follow-up period; however, one patient had a case of herpes during the follow-up period.

The four DSO cases included four female subjects with a mean age of 64.2 ± 9.7 years. Only one eye was operated on from each patient. Average values, standard deviations (SDs), and ranges for the various parameters at the preoperative and postoperative stages are shown in Table 2.

**Figure 3:**
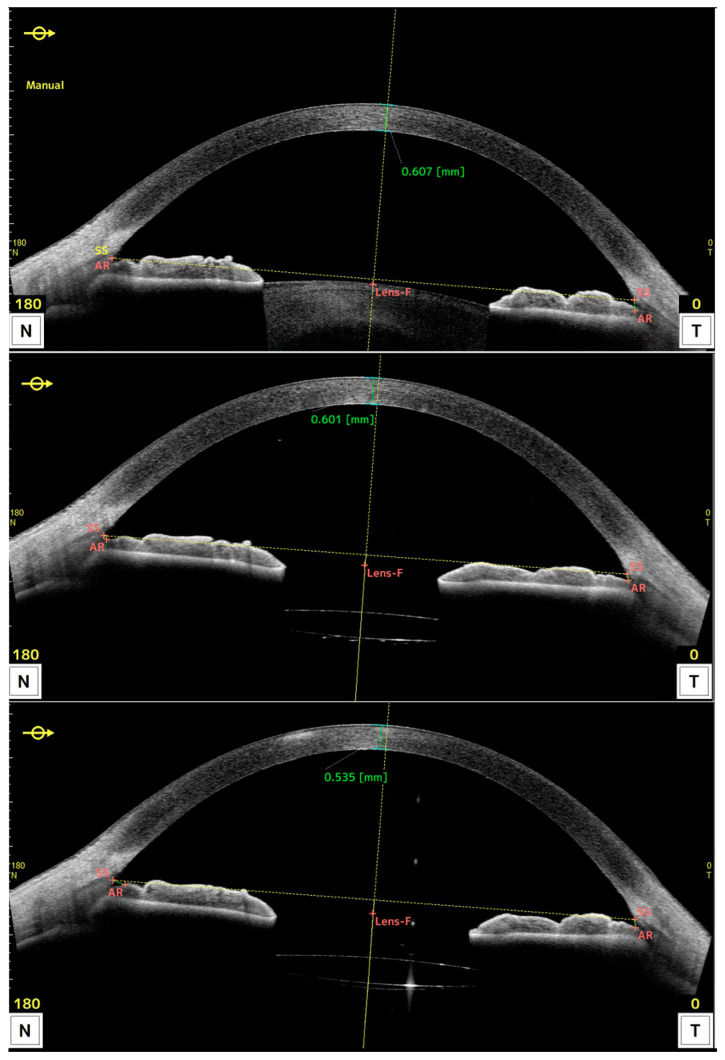
OCT along the horizontal meridian obtained preoperatively (**upper**), and 1 month (**middle**) and 3 months (**lower**) after DSO surgery for one patient. Values shown in green refer to central pachymetry.

Figure 3 shows the OCT obtained preoperatively (upper), and 1 month (middle) and 3 months (lower) after DSO surgery for one patient, displaying central pachymetry values in green.

The average improvement in the CDVA 3 months after surgery compared with prior to surgery was 0.09 ± 0.17 decimal. All cases had a phacoemulsification procedure carried out as well. The average change in cell count between 1 month and 3 months after the surgery was 460 ± 515.69 cells/mm^2^ (cell count was not obtained in the 1 month visit for one of the patients). There were no associated complications during the surgery or the follow-up period, but irregular astigmatism was identified in one patient, and another patient developed stromal fibrosis.

## 6. Discussion

This is, to the best of our knowledge, the first study to describe and report outcomes of DSO surgery using the NGENUITY^®^ 3D visualization system; unfortunately, no comparison with previous clinical studies is possible. However, in relation to DMEK surgery, only three previous studies have been published comparing the NGENUITY^®^ system with a conventional operating microscope: the OPMI-Lumera 700. Panthier et al. [7] reported in 2021 the outcomes of 12 cases of DMEK surgery using the NGENUITY^®^ system to assess the feasibility of this system in this surgery and to compare with standard DMEK procedure utilizing the OPMI-Lumera 700. They recorded some parameters such as DMEK graft preparation time, graft unfolding time, time to perform the DM, and the overall surgical time, in addition to standard metrics such as CDVA, endothelial cell density of the donor tissue, and the recipient’s central corneal thickness both before and at 1 and 3 months postsurgery. Their results revealed that the time to perform the graft preparation, DM, and overall surgical time were significantly higher with the NGENUITY^®^ system than with the conventional operating microscope; the time to unfold the graft was also higher, although not significantly, in the NGENUITY^®^ system group compared with the conventional group (*p* = 0.89). The mean central corneal thickness decreased in both groups at 3 months after surgery without a significant difference (*p* = 0.60). Additionally, the CDVA was similar in both groups with no significant differences, and the percentage of endothelial loss was also similar (about 30%) without a significant difference (*p* > 0.05). These authors concluded that performing this type of surgery using the NGENUITY^®^ system is feasible during some steps of the surgery (i.e., correct position determination of the Descemet membrane roll), but they found it was more challenging, with the total time being longer compared with using a conventional microscope. One important benefit of this procedure is that it allows the surgical assistance and operating room staff to share the same view as the surgeons; therefore, this is a step forward for instructional courses.

A recent paper published by Borroni et al. [8] evaluated the feasibility of DMEK tissue preparation using the NGENUITY^®^ system and compared it with the OPMI-Lumera 700. They selected 28 healthy pairs of corneas and randomly divided them into two groups. Each pair of corneas had one cornea prepared with NGENUITY^®^ with a 5 s staining time using VisionBlue™, and the other cornea was prepared using the conventional surgical microscope with a 30 s staining time. They evaluated DMEK graft preparation time, speed of stripping, graft width, and endothelial cell loss. Their results revealed that the graft preparation time was significantly higher using the NGENUITY^®^ system compared with using the conventional one (*p* = 0.031); the mean of speed of stripping was also higher for the NGENUITY^®^ system (*p* = 0.024). The mean endothelial cell density after tissue preparation was reduced in both group (*p* > 0.05), and the graft width was no different between them (*p* > 0.05). These authors concluded that DMEK tissue preparation using the NGENUITY^®^ system was feasible, with a slightly increased preparation time; the improved visualization allowed a reduced staining time that could be beneficial for eye banks due to the possible reduction in the staining colorants’ toxic effect.

Recently, Morelli et al. [9] also compared both systems in a sample of 20 eyes operated with the NGENUITY^®^ and 20 eyes using the OPMI-Lumera 700. All of the patients in this study were affected by FECD. Global surgical time, time to perform the DM, CDVA, central corneal thickness, endothelial cell density, and corneal densitometry were measured before and at 1, 3, and 6 months postsurgery. Their results revealed that the global surgical time and time to perform DM were significantly lower in the traditional microscope group (*p* = 0.04 and *p* = 0.02, respectively) and that the CDVA, central corneal thickness, endothelial cell density, and corneal densitometry values did not differ significantly in the two groups at all follow-ups (*p* > 0.05). They concluded that the use of the NGENUITY^®^ system can be employed in DMEK surgery based on the satisfactory clinical outcomes obtained; however, the slightly longer surgical time of the 3D DMEK surgeries may lead to hesitancy in surgeons. They considered that the main advantages of the NGENUITY^®^ system approach may be the improved ergonomic comfort during surgery and the utility of assistants in surgical training.

Although not specifically analyzed in the present study, the authors confirm previous reports of substantial ergonomic benefits of 3D visualization for surgeons [10,11], advantages in real time, in-theater simultaneous viewing of the surgery by other surgeons and/or staff, expanding the possibilities with regard to training, and didactic purposes [11,12,13,14]. The learning curve was very short, as, in the present case, there was no training for the authors prior to this study.

The outcomes of the present study confirm the findings reported by others with regard to the use of 3D visualization during DMEK surgery, with outcomes that are not worse than conventional surgery and without associated complications, as well as successfully expanding applicability to DSO surgery without any complication compared with the conventional procedure.

As a limitation of this work, the surgeon’s learning curve was not studied. This work is a brief report carried out without analysis of surgical times, without study of the ergonomic advantages of the system, and without a control group using traditional microscopy.

## Figures and Tables

**Table 1:** Average values and ranges for the different parameters measured in the DMEK patients.

Presurgery	Average ± SD	Range
Pachymetry (μm)	636.80 ± 67.01	558–735
CDVA (decimal)	0.35 ± 0.09	0.2–0.45
1 month Postoperation		
Pachymetry (μm)	578.67 ± 319.59	538–645
CDVA (decimal)	0.77 ± 0.14	0.6–0.95
Endothelial cell count (cells/mm^2^)	1724 ± 849.08	1209–2181
3 months Postoperation		
Pachymetry (μm)	490.67 ± 270.94	436–529
CDVA (decimal)	0.81 ± 0.19	0.50–1
Endothelial cell count (cells/mm^2^)	2112 ± 279.80	1621–2315

**Table 2:** Average values and ranges for the different parameters measured in the DSO patients.

Presurgery	Average ± SD	Range
Pachymetry (μm)	625.00 ± 117.64	551–800
CDVA (decimal)	0.65 ± 0.19	0.4–0.8
1 month Postoperation		
Pachymetry (μm)	622.50 ± 93.84	553–755
CDVA (decimal)	0.65 ± 0.29	0.3–1.0
Endothelial cell count (cells/mm^2^)	602.00 ± 301.00	602–897
3 months Postoperation		
Pachymetry (μm)	549.50 ± 36.38	521–601
CDVA (decimal)	0.74 ± 0.30	0.45–1
Endothelial cell count (cells/mm^2^)	1249 ± 428.08	671–1640

## Data Availability

Data is available from the authors upon reasonable request.
